# A TFAIII-Type Transcription Factor *OsZFPH* Regulating a Signaling Pathway Confers Resistance to *Xanthomonas oryzae* pv. *Oryzae* in Rice

**DOI:** 10.3390/genes16030240

**Published:** 2025-02-20

**Authors:** Chunyun Yang, Xinxiang A, Cuifeng Tang, Chao Dong, Feifei Zhang, Chunmei He, Yiding Sun, Yi Yang, Sandan Yan, Yanhong Liu, Yayun Yang, Luyuan Dai

**Affiliations:** 1 Institute of Biotechnology and Germplasm Resources, Yunnan Academy of Agricultural Sciences, Yunnan Provincial Key Laboratory of Agricultural Biotechnology, Key Laboratory of Southwestern Crop Gene Resources and Germplasm Innovation, Scientific Observation Station for Rice Germplasm Resources of Yunnan, Ministry of Agriculture and Rural Affairs, Kunming 650205, China; ycy1451@163.com (C.Y.); ahope2002@163.com (X.A.); tangcuif@163.com (C.T.); cchaodong@126.com (C.D.); awennazhang@163.com (F.Z.); 18987967684@163.com (C.H.); yidingsun18@163.com (Y.S.); xiaoyang0326@126.com (Y.Y.); 2 Xishuangbanna Agricultural Science Research Institute, Jinghong 666100, China; yansandan@126.com; 3 Agricultural Environmental Protection and Rural Energy Workstation, Yuanjiang 653300, China; yjxgov@163.com; 4 Yunnan Academy of Agricultural Sciences, Kunming 650205, China; luyuandai@163.com

**Keywords:** local rice, Haonuoyang, bacterial leaf blight (BLB), TFAIII-type transcription factor, *OsZFPH*, gene cloning, functional analysis

## Abstract

Background: Rice bacterial leaf blight, caused by the Gram-negative bacterium *Xanthomonas oryzae* pv. *Oryzae* (*Xoo*), significantly impacts rice production. To address this disease, research efforts have focused on discovering and utilizing novel disease-resistant genes and examining their functional mechanisms. Methods and Results: In this study, a variety of bacterial strains were utilized. CX28-3, AX-11, JC12-2, and X10 were isolated from the high-altitude japonica rice-growing region on the Yunnan Plateau. Additionally, PXO61, PXO86, PXO99, and PXO339, sourced from the International Rice Research Institute (IRRI), were included in the analysis. To evaluate the resistance characteristics of Haonuoyang, artificial leaf cutting and inoculation methods were applied. Results indicated that Haonuoyang exhibited broad-spectrum resistance. Additionally, to explore the genetic mechanisms of resistance, the TFAIII-type transcription factor *OsZFPH* was cloned from Haonuoyang using PCR amplification. The subcellular localization method identified the precise location of the *OsZFPH* gene within the cell. The expression of *OsZFPH* was induced by *Xoo* stress. The overexpression of *OsZFPH* resulted in increased activities of enzymes, including SOD, CAT, and POD, while silencing the gene led to reduced enzyme activities. Furthermore, the hormones SA (salicylic acid), JA (jasmonic acid), and GA (gibberellin) were shown to positively regulate the gene expression. Protein interactions with *OsZFPH* were verified through a yeast two-hybrid system and BiFC technology. Hap5, which aligned with the sequence of Haonuoyang, was found to belong to a haplotype consisting of Jingang 30, 40 resequenced rice varieties, 18 *Oryza rufipogon*, and 29 *Oryza granulata*. Conclusions: The findings of this study emphasize the vital role of *OsZFPH* in rice resistance to bacterial leaf blight. The identification of broad-spectrum resistance in Haonuoyang and the understanding of *OsZFPH* gene functions provide valuable insights for the future development of rice varieties with improved resistance to this destructive disease.

## 1. Introduction

Rice is a vital food crop around the world [1]. However, its yield growth rate has not kept up with the growing population. Bacterial leaf blight is one of the most severe bacterial diseases affecting rice globally. In extreme cases, it can cause a 50% reduction in yield or even result in complete crop failure. The Gram-negative bacterium *X. oryzae* pv. *Oryzae* (*Xoo*), responsible for bacterial leaf blight (BLB), is a significant economic threat to rice production worldwide [2]. Traditional chemical methods used to control BLB have proven ineffective, costly, and harmful to the environment. Historically, hybrid breeding was the main method for introducing disease-resistant genes. However, as the pathogen evolved, varieties that initially showed strong resistance gradually lost it. The most cost-effective approach has been to develop and utilize disease-resistant varieties [3].

With the advancement of molecular biology techniques, new genes conferring resistance to leaf blight are continually being discovered, characterized, and cloned. Over 49 resistance genes or loci for BLB have been identified so far, and these genes are located on chromosomes other than chromosomes 9 and 10. Seven genes remain unlocalized (xa15, Xa16, Xa17, Xa18, xa26(t), xa28(t), Xa37(t)), while the others have been mapped. Notably, Xa1 [4], Xa2, Xa3/Xa26, Xa4, xa5 [5], Xa10, xa13 [6], Xa14, Xa21, Xa23, and an additional 17 genes, including 13 dominant and four recessive genes, have been successfully cloned [7]. Many of these genes, which provide strong resistance to leaf blight, originate from wild rice. Widely used genes, such as Xa1, Xa3 [8], Xa4 [9], *xa5* [10], Xa7 [11], and xa13 [12], are known to be effective, but their resistance is vulnerable to overcoming due to the limitations of a single resistance gene and a narrow spectrum of resistance. Xa21 [13,14] is currently the most extensively used gene due to its broad-spectrum, durable resistance. It encodes a receptor-like protein kinase and contains three key structural features commonly found in other resistance genes: Xa21 is distinctive among plant resistance genes because it possesses leucine-rich repeat (LRR) extracellular, transmembrane, and cytoplasmic serine and threonine kinase domains. Additionally, the Xa23 gene [15,16], a novel resistance gene derived from wild rice (O. rufipogon), has gained increasing attention. This gene targets transcription activator-like effectors (TALEs) from pathogens, triggering a strong defense response and hypersensitive reaction in the host, which offers exceptional resistance to BLB. It encodes a 113-amino acid protein.

The regulation of disease and stress resistance is essential in rice breeding and disease management, making TFIIIA-type zinc finger proteins in plants a key area of research. These proteins, which belong to the C_2_H_2_-type zinc finger family, are vital regulatory proteins with nucleic acid-binding functions. They are the most widely distributed type in plants and play a role in regulating a variety of physiological and biochemical processes. For instance, they can control plant disease resistance and stress tolerance by binding to specific DNA sequences and interacting with RNA [17,18]. Most research on C_2_H_2_-type zinc finger proteins has focused on their role in response to stress conditions like drought, low temperature, and salinity. There is a complex signal transduction network through which hormones and C_2_H_2_-type zinc finger proteins interact. For example, the zinc finger protein ZFP207 (Cys2/His2 type) negatively regulates GA synthesis by binding to the *OsGA20ox2* (also known as the Green Revolution Gene *SD1*) promoter, thereby inhibiting its expression and resulting in dwarfism and shortened grain and panicle phenotypes in rice [19]. In the jasmonic acid (JA) signaling pathway, JA-related proteins interact with specific C_2_H_2_-type zinc finger proteins, altering their conformation or location and enhancing their transcriptional regulation. These proteins then work together to modulate the plant’s defense against diseases and other critical physiological processes. For instance, *OsDOF24* is induced by methyl jasmonate (JA), which subsequently inhibits JA biosynthesis by directly binding to the promoter of the JA biosynthetic gene *OsAOS1*, likely suppressing its expression and influencing leaf senescence [20]. C_2_H_2_-type zinc finger proteins may also regulate the expression of genes involved in SA synthesis and metabolism. They can bind to the promoters of key SA synthesis genes such as ICS1 to enhance their expression and boost SA production. Alternatively, they may interact with SA metabolism genes to regulate the degradation rate of SA, helping maintain a balance of SA in plants under varying conditions [21]. MoSDT1, a C_2_H_2_ zinc effector, activates the rice defense system while increasing the pathogenicity of the rice blast fungus [22]. However, the role of these proteins in influencing disease resistance has been inadequately studied. It is therefore important to identify new C_2_H_2_-type zinc finger proteins and explore their functions.

In this study, the artificial leaf cutting inoculation method was used to assess disease resistance. Bacterial strains CX28-3, AX-11, JC12-2, X10, PXO61, PXO86, PXO99, PXO112, and PXO339 were inoculated onto Haonuoyang, which is a local rice variety from Yunnan. Haonuoyang was found to possess broad-spectrum resistance. It was hypothesized that Haonuoyang carries a bacterial leaf blight resistance gene, which was tentatively named OsZFPH. The gene *OsZFPH* was cloned, and its antioxidant enzyme activity, expression patterns, resistance levels, hormone signaling pathways, and subcellular localization were analyzed. The aim of this study was to better understand the role of OsZFPH in the resistance and defense mechanisms against bacterial leaf blight and to contribute to the broader understanding of C_2_H_2_-type zinc finger proteins in the resistance of rice to bacterial leaf blight and their underlying molecular mechanisms.

## 2. Materials and Methods

### 2.1. Experimental Material

In this study, a broad selection of rice materials was utilized, including Haonuoyang (HNY), Jingang 30 (G30), Zhenzhu’ai, and Nipponbare (NP). *OsZFPH* overexpression plants with Nipponbare as the background (H1) and *OsZFPH* knockout plants with Haonuoyang as the background (Z1) were also employed for genetic analysis. The study incorporated 40 resequenced accessions of both indica and japonica rice to investigate the genetic diversity associated with disease resistance. Additionally, 15 accessions of *Oryza officinalis* Wall, 29 accessions of *O. granulata* Nees, and 19 accessions of *O. rufipogon* Griff were included to examine the origin and evolution of potential disease-resistance genes. Highly virulent *X. oryzae* pv. *Oryzae* (*Xoo*) strains were selected for the study, including CX28-3, AX-11, JC12-2, and X10, which were isolated from highland japonica rice. These local strains were valuable as they represent the unique pathogen population found in high-altitude regions. Moreover, PXO61, PXO86, PXO99, PXO112, and PXO339 strains from the International Rice Research Institute were included to serve as a standard benchmark for comparing the virulence and resistance responses of the rice materials. The primers for the genes involved in the experiment are provided in Table 1 below.

### 2.2. Rice Plants and Methods of Pathogen Inoculation and Investigation

The rice seeds were immersed in a 15% sodium hypochlorite solution for 20 to 30 min. Afterward, the seeds were thoroughly rinsed with distilled water and placed in a Petri dish. A sufficient amount of water was added, and the seeds were kept at room temperature until they germinated. Once germination occurred, the seeds were transferred to the Songming Scientific Research Base of Yunnan Academy of Agricultural Sciences. Inoculation preparation was initiated when the rice reached the booting stage. The *Xoo* was preserved in glycerol at −80 °C. Upon retrieval, the bacteria were dispersed and inoculated onto NA medium, which contained 3 g/L beef extract, 5 g/L peptone, 2.5 g/L glucose, and 18 g/L agar powder. The pH was adjusted to 7–7.2, and the bacteria were rejuvenated in an incubator set at 28 °C. The bacterial culture was streaked and subcultured twice. A bacterial suspension of 3 × 10^8^ CFU·mL^−1^ (with an optical density at D600 nm about 0.5) was prepared for inoculation. The leaf-cutting inoculation was performed within one hour [17]. Following inoculation, the rice plants were placed in an environment with a temperature between 28 and 30 °C for growth. Samples were collected at specified time points, and the disease incidence was evaluated after 21 days. The full length and lesion length of 5–7 inoculated leaves from each plant were measured, and the average percentage of lesion length for each plant was calculated.

### 2.3. Henotypic Verification of OsZFPH Transgenic Plants via PXO99 Inoculation

A total of 175 plants from 25 lines of T_3_ generation transgenic rice with RNA interference targeting the CDS region of the *OsZFPH* gene in the Haonuoyang variety (derived from Haonuoyang transformation) and 23 T_1_ generation transgenic plants with overexpression of the *OsZFPH* gene (derived from Nipponbare transformation) were successfully obtained. The T_3_ generation transgenic plants with RNA interference, which were confirmed positive through PCR detection, were cultivated at the Gasa base in Xishuangbanna. The T_1_ generation overexpression transgenic plants were grown in a greenhouse at the Gasa base. Leaf-cutting inoculation was then performed on each plant using PXO99 with five leaves inoculated per plant. Lesion lengths were measured, and the average lesion percentage was calculated as per the method outlined in Section 2.2.

### 2.4. DNA Extraction and Real-Time Fluorescent Quantitative PCR

Leaves were collected from the plants, and DNA was extracted from the flag leaves using the CTAB method. For RNA extraction, 50–100 mg of the samples was weighed, thoroughly ground with liquid nitrogen, and the total RNA was extracted using the EastepTM Total RNA Super Extraction Kit (Promega, Beijing, China). The HiFiScript cDNA Synthesis Kit (CWBIO, Beijing, China) was used for cDNA synthesis, following the manufacturer’s guidelines. Real-time fluorescence quantitative PCR analysis was performed using the *OsActin* gene as the internal control, and the experiment was conducted in triplicate. The relative gene expression levels were calculated using the 2^−ΔΔCt^ method.

### 2.5. Determination of H_2_O_2_ Content and Antioxidant Enzyme Activities in OsZFPH Transgenic Plants

The kits for measuring H_2_O_2_ content, POD, SOD, and CAT activities were obtained from Sangon, Shanghai, China ( (H_2_O_2_: D799774-0100, POD: D799592-0100, SOD: D799594-0100, CAT: D799598-0100). The procedures were carried out according to the provided instructions.

### 2.6. Analysis of the Relationship Between Exogenous Hormones and OsZFPH Gene Expression

Haonuoyang (which contains the *OsZFPH* gene) and Jingang 30 (without this gene) were used as experimental materials. Both were treated with 100 μmol/L methyl jasmonate (JA), 100 mmol/L salicylic acid (SA), and 100 mmol/L gibberellin (GA), with Jingang 30 as the control. Samples were collected at 0 h, 4 h, 8 h, 12 h, 24 h, 48 h, and 72 h, and the expression of *OsZFPH* was quantified using real-time PCR.

### 2.7. Transient Expression and Subcellular Localization of OsZFPH Gene in Tobacco Leaves

Tobacco seeds were sown and cultivated under a controlled 12 h photoperiod for one month, after which the seedlings were considered ready for experimentation. The preconstructed vector plasmid was introduced into *Agrobacterium tumefaciens* strain GV3101 using electroporation. Following transformation, the *Agrobacterium* GV3101 was incubated at 30 °C for two days. Using an inoculation loop, the *Agrobacterium* colonies were carefully scraped from the surface of the agar plate and transferred to 10 mL of YEB liquid medium. The medium was then shaken at 170 rpm for one hour. Afterward, the culture was centrifuged at 4000 rpm for four minutes, and the supernatant was discarded. The bacterial pellet was resuspended in a 10 mM magnesium chloride (MgCl_2_) solution containing 120 μM acetosyringone (AS). The optical density (OD600) of the resuspended bacterial solution was adjusted to about 0.6. Healthy tobacco plants were chosen for the injection process. A 1 mL syringe with the tip removed was used to inject the bacterial solution into the lower epidermis of the tobacco leaves. Each injection site was marked for tracking purposes. The plants were kept under low-light conditions for two days post-injection. After this period, the tobacco leaves that had been injected with the labeled *Agrobacterium* were collected, processed into slides, and examined under a laser-scanning confocal microscope. High-resolution images were captured during the observation.

### 2.8. Screening Proteins Interacting with OsZFPH

#### 2.8.1. Yeast Two-Hybrid Assay

In this study, Haonuoyang rice and *Nicotiana benthamiana* were selected to investigate plant–pathogen interactions and gene functions. The total RNA was extracted using the EastepTM kit (Promega, Shanghai, China). cDNA synthesis, essential for gene amplification, was performed with the HiFiScript Kit (CWBIO, Beijing, China). PCR amplification was conducted using Vazyme’s 2 × Phanta Max Master Mix (Vazyme, Nanjing, China) to ensure high accuracy. DNA fragments were purified with Sangon Biotech’s gel extraction kit (Sangon, Shanghai, China). DH5α competent cells, supplied by the same company, facilitated DNA transformation. Recombinant plasmids were constructed using Beyotime’s seamless cloning kit (Beyotime, Shanghai, China), while DNA digestion was carried out with TaKaRa’s restriction endonucleases. Plasmid DNA was subsequently isolated with the SanPrep kit (Sangon, Shanghai, China). Lastly, yeast two-hybrid assays for protein–protein interaction analysis were conducted using Vazyland’s Y2HGold cells (Vazyme, Nanjing, China)

#### 2.8.2. Bimolecular Fluorescence Complementation (BiFC) Technique

Total RNA from rice and tobacco was extracted using the EastepTM kit and subsequently reverse-transcribed into cDNA with the HiFiScript Kit (CWBIO, Beijing, China). Primers specific to *OsZFPH* and interacting protein genes were designed and amplified using Vazyme’s 2×Phanta Max. The resulting fragments were purified with Sangon’s gel extraction kit (Sangon, Shanghai, China). The pFGC-YC155 and pFGC-YN173 vectors were digested with TaKaRa’s endonucleases. Gene fragments were ligated using Beyotime’s kit and transformed into DH5α cells. Kanamycin-containing plates were used for screening, and plasmid construction was verified through sequencing. Recombinant plasmids were then transferred into GV3101 cells, and positive clones were selected using kanamycin-containing plates. Following incubation of the injected tobacco plants in a growth chamber at 25–28 °C for 2–3 days, fluorescence signals in the injected leaf regions were observed under a fluorescence microscope.

### 2.9. Haplotype Analysis of the Transcription Factor OsZFPH

A total of 104 materials exhibiting resistant and susceptible phenotype differentiation, including 40 indica and japonica rice (resequenced rice), 15 *O. officinalis*, 29 *O. granulata*, 18 *O. rufipogon*, Haonuoyang, and Jingang 30, were analyzed for homologous *OsZFPH* genes. PCR amplification was performed using gene-specific primers 5′-ATGGAGGAGCAAAGAGGCGA-3′ and 5′-TAAGCGTGCACAACAGCTT-3′. Gene cloning was conducted using RACE technology. Additionally, *OsZFPH* genes from 104 rice materials were inserted into the pMD18-T vector. Sequencing results were subsequently used for haplotype analysis. MEGA11 was employed for sequence analysis.

### 2.10. Statistical Analyses

All data were analyzed using DPS data analysis software v15.10 and are presented as mean ± SD. Statistically significant differences (*p* < 0.05) are indicated by different letters.

## 3. Results

### 3.1. Characterization of Yunnan Local Rice HNY for High Resistance to Bacterial Leaf Blight

**Figure 1:**
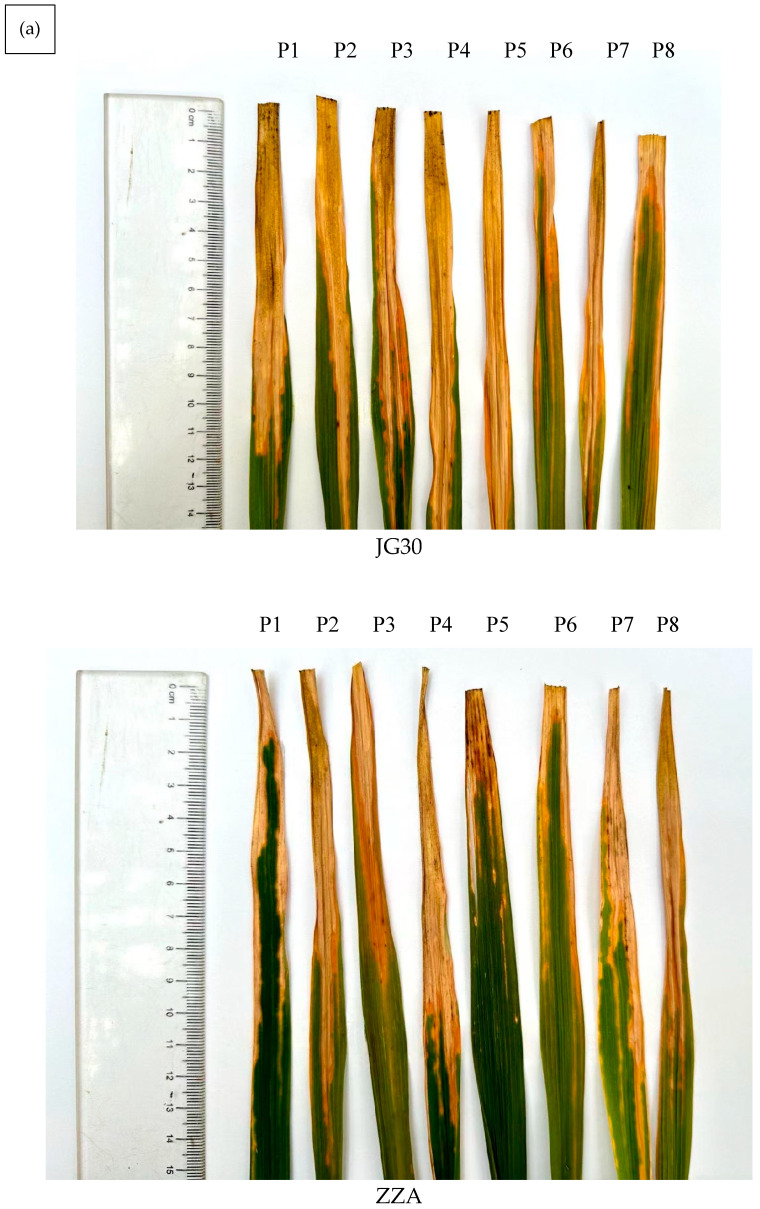

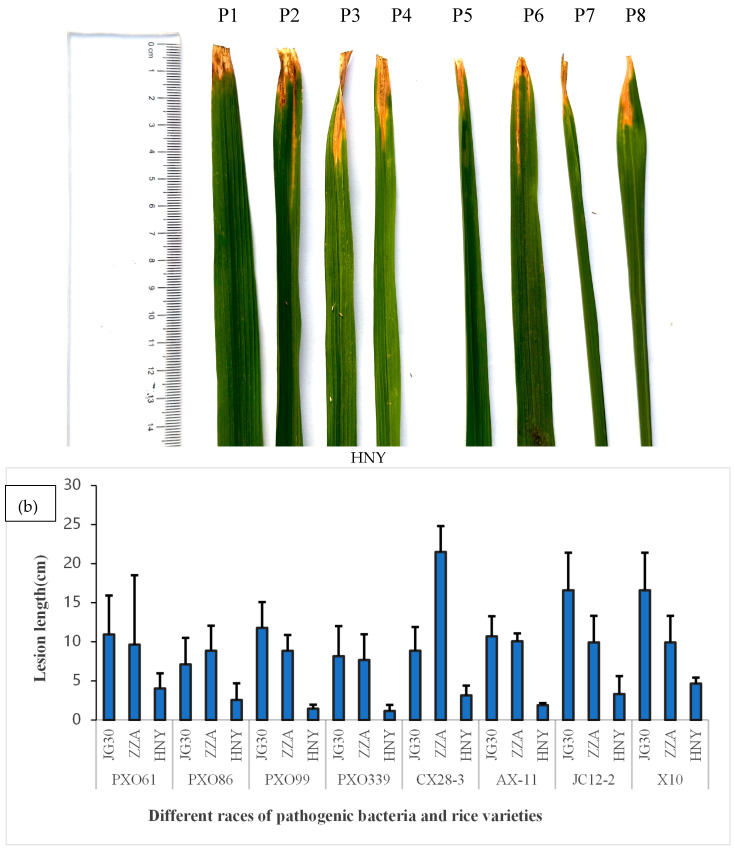
Lesion lengths in Haonuoyang and the susceptible rice varieties Jingang 30 and Zhenzhu’ai after leaf-clipping inoculation with eight races of *Xoo*. The eight *Xoo* races are P1 (PXO61), P2 (PXO86), P3 (PXO99), P4 (PXO339), P5 (CX28-3), P6 (AX-11), P7 (JC12-2), and P8 (X10). (**a**) The lesions of three rice materials after being infected by eight *Xoo* isolates. (**b**) The average lesion lengths of three rice materials after being infected by eight *Xoo* strains. HNY: Haonuoyang; JG30: Jinggang30; ZZA: Zhenzhuai.

Following inoculation with the international race-differentiating strains PXO61, PXO86, PXO99, PXO339, CX28-3, AX-11, JC12-2, and X10, Jingang 30 and Zhenzhu’ai exhibited high susceptibility to *Xoo*, whereas Haonuoyang demonstrated moderate to high resistance (Figure 1a,b).

### 3.2. Acquisition of OsZFPH: A TFAIII Transcription Factor Gene of Yunnan Local Rice HNY

**Figure 2:**
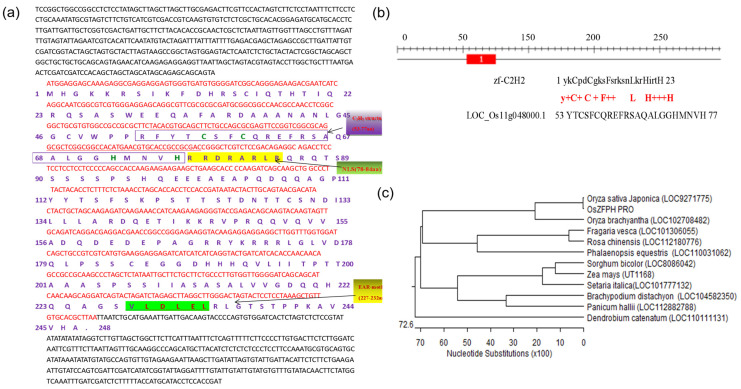
Amino acid sequence, functional prediction, and phylogenetic analysis of *OsZFPH*. (**a**) Full-length cDNA sequence of *OsZFPH* (underlined region indicates the C_2_H_2_ zinc finger structural domain). The structure marked by the purple box is the C_2_H_2_ structure, 53aa-77aa; the area indicated by the green box is the nuclear localization signal (NLS) region, 77aa-84aa; the structure marked by the yellow box is the EAR-motif, 227aa-232aa. (**b**) Functional domain prediction of the *OsZFPH* coding sequence (CDS)-encoded amino acid sequence. Generally, the typical structure of zf-C_2_H_2_ is ‘ykCpdCgksFsrksnLkrHirtH’, which consists of 23 amino acids. The C_2_H_2_ structure of *OsZFPH* (LOC_Os11g048000.1) was ‘YTCSFCQREFRSAQALGGHMNVH’. (**c**) Phylogenetic tree of the *OsZFPH*-encoded amino acid sequence compared with homologous sequences from other species. Red marked the ORF region of the gene.

Based on sequence prediction conducted on the NCBI website, specific primers (F: 5′ATGGAGGAGCAAAGAGGCGA3′; R: 5′TAAGCGTGCACAACAGCTT3′) were designed. Subsequently, the *OsZFPH* gene, encoding a TFAIII-type (C_2_H_2_-type) zinc-finger-structured protein, was successfully cloned from Haonuoyang. In accordance with the rice gene nomenclature system, this gene was designated *Oryza sativa* Zinc Finger Protein Haonuoyang (*OsZFPH*) and registered on the Rice Gene Online Submission website. The complete coding DNA sequence (CDS) of this gene consists of 728 base pairs (bp) and encodes a polypeptide comprising 243 amino acid residues. The C_2_H_2_ structural domain was computationally predicted to be positioned between the 53rd and 77th amino acid residues, encompassing the characteristic QALGGH motif specific to zinc-finger proteins. Additionally, the gene contains a nuclear localization signal region (RRDRAKLR, residues 77–84) and an EAR-motif transcriptional regulatory region (VLDLEL, residues 227–232), whereas no L-box region was identified (as shown in Figure 2a,b). Evolutionary analysis of the *OsZFPH* gene across rice and other species revealed that *OsZFPH* from this rice variety shares 99.86% sequence similarity with the transcription factor RIBBIT EARS (LOC9271775) of indica rice (Figure 2c).

### 3.3. Phenotypic Verification via Inoculating OsZFPH Transgenic Plants with PXO99

**Figure 3:**
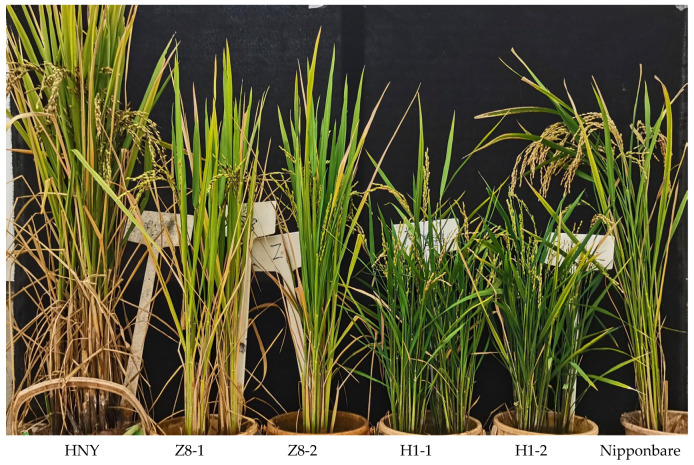
Phenotypic validation of different *OsZFPH* transgenic rice plants.

**Figure 4:**
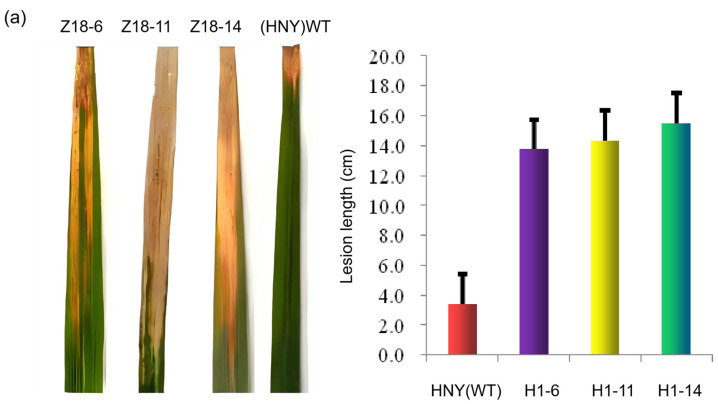

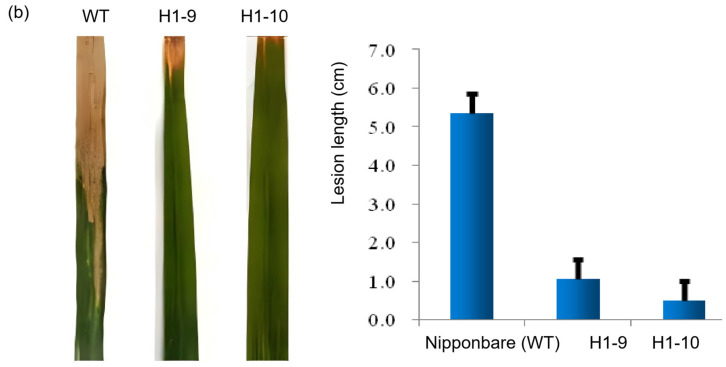
Lesion lengths in RNAi transgenic plants (T_3_ generation) and overexpression transgenic plants (T_2_ generation) after inoculation with PXO99. (**a**) Lesion lengths in RNAi transgenic plants (T_3_ generation) after inoculation with PXO99; (**b**) Lesion lengths in overexpression transgenic plants (T_2_ generation) after inoculation with PXO99.H1 represents the overexpression transgenic plant line for *OsZFPH*, Z8 represents the RNAi transgenic plant line for *OsZFPH*, HNY (WT) represents the wild-type Haonuoyang, and Nipponbare (WT) represents the wild-type Nipponbare.

From the perspective of plant phenotypes, plants with overexpressed and knocked-out *OsZFPH* genes exhibited shorter stature compared to wild-type HNY plants. The average tiller number in overexpressing plants reached 30, which was nearly twice that observed in knocked-out plants (13), wild-type HNY plants (15), and Nipponbare (14) (Figure 3). A total of 175 plants from 25 RNA-interfering T_3_ generation transgenic lines (transformed HNY) grown at Gasa base in Xishuangbanna, along with 23 T_1_ generation transgenic plants overexpressing the *OsZFPH* gene at Songming base of Yunnan Academy of Agricultural Sciences, were inoculated with PXO99. Following inoculation, the average lesion length in RNA-interfering transgenic plants was recorded as 10.1 cm ±1.3, indicating susceptibility, while the wild-type HNY plants exhibited an average lesion length of 3.4 cm ± 0.3, signifying disease resistance. Additionally, 23 T_2_ generation transgenic plants overexpressing *OsZFPH* were inoculated with PXO99, where the average lesion length of overexpressing plants was 2.0 cm ± 1.5, indicating resistance. In contrast, the control Nipponbare (CK1) exhibited an average lesion length of 6.3 cm ± 0.5, indicating susceptibility (Figure 4). Based on the combined results of disease resistance phenotypes in transgenic plants, all RNA-interfering transgenic lines exhibited moderate susceptibility to susceptibility toward PXO99, whereas all overexpressing lines demonstrated resistance to PXO99.

### 3.4. The Expression of the OsZFPH Gene Is Triggered in Response to PXO99

**Figure 5:**
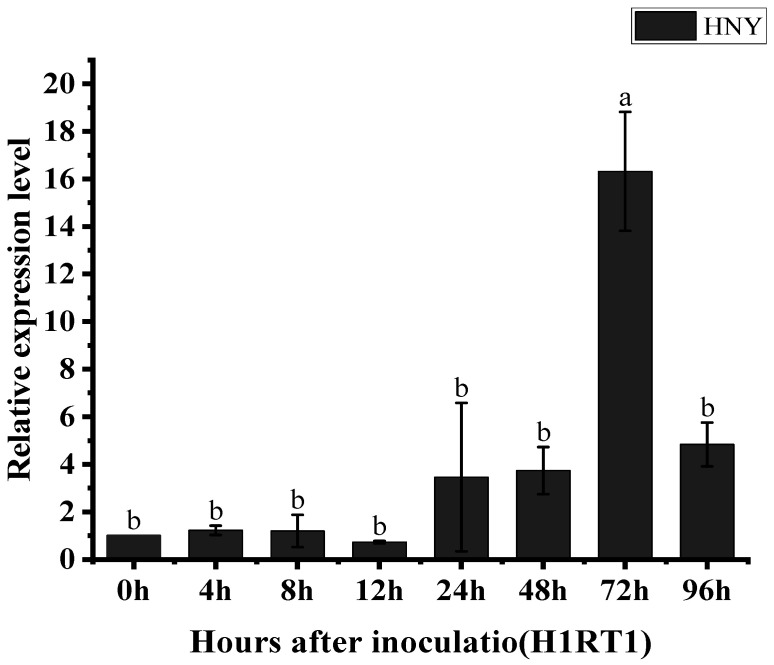
Expression of *OsZFPH* at different time points after PXO99 infection in HNY. Error bars indicate standard deviation (SD), and different letters denote statistically significant differences (*p* < 0.05).

To determine whether the expression of the *OsZFPH* gene is induced by stress caused by *X. oryzae* pv. *oryzae* (*Xoo*), the causal agent of bacterial blight, gene-specific primers for quantitative real-time polymerase chain reaction (qRT-PCR) were carefully designed. The rice cultivar Haonuoyang was inoculated with the *Xoo* strain PXO99, and samples were collected at 0 h, 4 h, 8 h, 12 h, 24 h, 48 h, 72 h, and 96 h post-inoculation. The expression profile of *OsZFPH* was analyzed using real-time fluorescence-based qRT-PCR assays. The results indicated that during the early stages of PXO99 infection (0 h, 4 h, 8 h, and 12 h), *OsZFPH* transcript levels remained relatively low. A notable upregulation was observed after 24 h, marking the initial peak. Expression continued to increase, reaching its highest level at 72 h, which was followed by a decline at 96 h (Figure 5). Overall, the temporal expression pattern of *OsZFPH* followed a parabolic trend. These findings suggest that *OsZFPH* expression is induced by *Xoo*-induced stress and likely plays a critical role in rice defense mechanisms against bacterial blight disease.

### 3.5. Expression of OsZFPH Gene in Different Rice Materials After Infestation by PXO99

**Figure 6:**
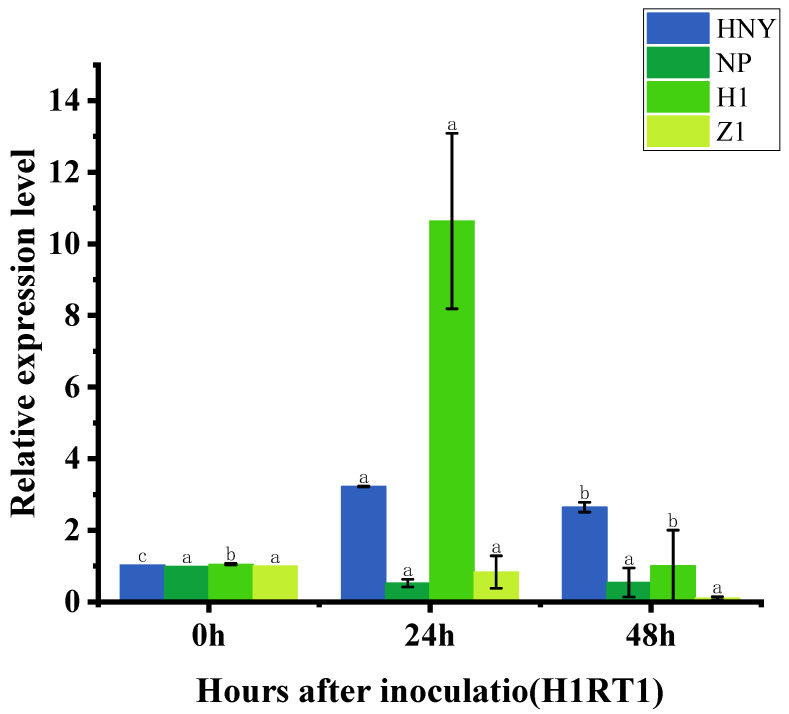
Expression of *OsZFPH* after PXO99 infiltration in different rice genotypes. Error bars indicate SD, and different letters denote statistically significant differences (*p* < 0.05).

Rice materials from different varieties were subjected to PXO99 stress for 0 h, 24 h, and 48 h. The results indicated that in H1, the relative expression level of the *OsZFPH* gene peaked at 24 h, reaching 10.64, which was higher than that observed in Haonuoyang (3.224), Nipponbare (0.528), and Z1 (0.84). In the gene-knockout material Z1, the relative expression level of *OsZFPH* exhibited a decreasing trend at 48 h (Figure 6).

### 3.6. OsZFPH Resistance Levels Analyzed in Relation to Hormone Signaling Levels

**Figure 7:**
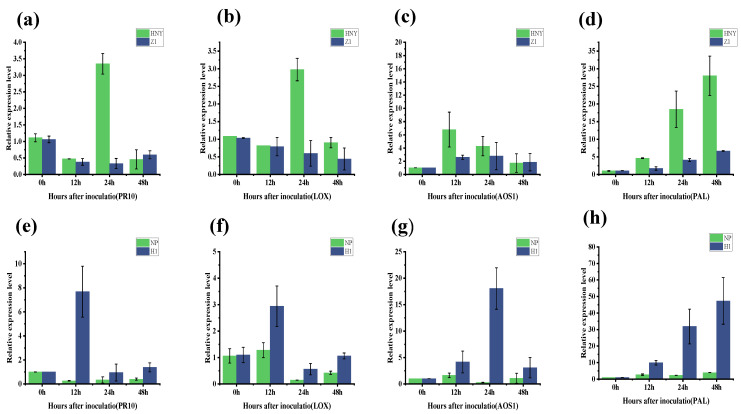
Expression of LOX, PAL, and AOS1 genes after PXO99 infection in different rice genotypes. (**a**) PR10 gene expression in HNY and Z1. (**b**) LOX gene expression in HNY and Z1. (**c**) AOS1 gene expression in HNY and Z1. (**d**) PAL gene expression in HNY and Z1. (**e**) PR10 gene expression in Nipponbare and H1. (**f**) LOX gene expression in Nipponbare and H1. (**g**) AOS1 gene expression in Nipponbare and H1. (**h**) PAL gene expression in Nipponbare and H1. Error bars indicate SD, and different letters denote statistically significant differences (*p* < 0.05).

Following inoculation with PXO99, the relative expression of the *PR10* gene in Haonuoyang peaked at 24 h. In contrast, no significant changes in PR10 gene expression were observed in Z1 when compared to Haonuoyang (Figure 7a). When H1 was compared with Nipponbare, the relative expression of the PR10 gene in H1 exhibited significant upregulation at 12 h (Figure 7e). The relative expression levels of key genes in the jasmonic acid (JA) pathway, namely LOX and AOS1, as well as the key gene in the salicylic acid (SA) pathway, PAL, were lower in Z1 than in Haonuoyang at 12 h, 24 h, and 48 h (Figure 7b–d). Additionally, these expression levels remained relatively unchanged over time. Conversely, in H1, the relative expression levels of the hormone-related genes LOX, PAL, and AOS1 were higher than those in Nipponbare at 12 h, 24 h, and 48 h (Figure 7f–h). These findings suggest that the *OsZFPH* gene contributed to systemic disease resistance and may have induced the expression of endogenous hormone-related genes to enhance the plant’s defense response.

### 3.7. Determination and Analysis of H_2_O_2_ Content and Antioxidant Enzyme Activities in Different OsZFPH-Harboring Plants Infected by PXO99

**Figure 8:**
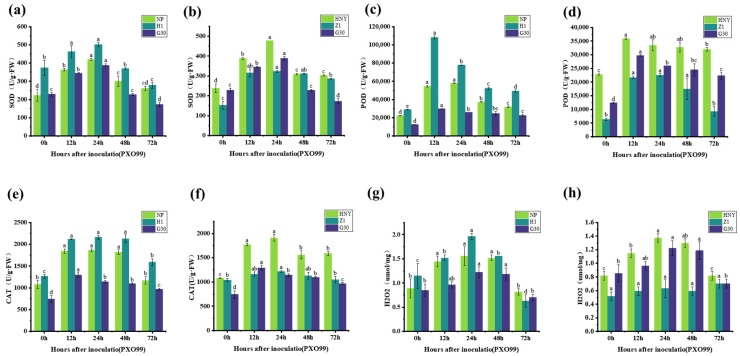
Changes in H_2_O_2_ content and antioxidant enzyme activities in *OsZFPH* transgenic and wild-type rice plants after PXO99 stress. (**a**) Superoxide dismutase (SOD) activity in Nipponbare, H1, and G30. (**b**) SOD activity in HNY, Z1, and G30. (**c**) Peroxidase (POD) activity in Nipponbare, H1, and G30. (**d**) POD activity in HNY, Z1, and G30. (**e**) Catalase (CAT) activity in Nipponbare, H1, and G30. (**f**) CAT activity in HNY, Z1, and G30. (**g**) H_2_O_2_ content in Nipponbare, H1, and G30. (**h**) H_2_O_2_ content in HNY, Z1, and G30. Error bars indicate SD, and different letters denote statistically significant differences (*p* < 0.05).

During the 0–72 h period following inoculation with PXO99, superoxide dismutase (SOD) activity in H1 exhibited an overall increasing trend, surpassing levels observed in both Nipponbare and JG30 (Figure 8a). In contrast, the SOD activity in Z1 and JG30 remained consistently lower than that in Haonuoyang throughout this timeframe (Figure 8b). In leaf tissues of H1, Z1, Haonuoyang, Nipponbare, and JG30, SOD activity followed a characteristic pattern of initial increase, peaking at 24 h, which was followed by a subsequent decline. A sharp rise was particularly evident at 12 h post-inoculation across all tested plant materials (Figure 8a,b). Upon infection with PXO99, SOD activity in Haonuoyang was significantly higher than that in Z1 and JG30, suggesting a stronger capability to mitigate reactive oxygen species (ROS)-induced damage. Similarly, SOD activity in H1 not only exceeded that in Nipponbare and JG30 but also surpassed that in Haonuoyang. These observations suggest that overexpression of the *OsZFPH* gene enhanced the ability of plants to counteract ROS-induced stress, possibly through the activation of additional defense pathways. An increase in the number of signal transduction factors associated with these pathways is also likely. Consequently, the overexpression of *OsZFPH* not only led to a significant upregulation of defense-related genes but also resulted in elevated SOD activity. This increase in SOD activity contributed to a stronger antioxidant defense mechanism, enhancing the plant’s overall resistance to pathogen-induced oxidative stress.

During the 0–72 h period following PXO99 inoculation, the peroxidase (POD) activity in H1, Z1, Haonuoyang, Nipponbare, and JG30 followed similar patterns. Initially, POD activity increased, which was followed by a decrease. It peaked at 12 h and began to decline between 12 and 24 h (Figure 8c,d). These findings suggest that PXO99 infection triggers an increase in POD activity. In general, POD activity in Haonuoyang was higher than in Z1 and JG30. Additionally, POD activity in H1 surpassed that in Nipponbare and JG30, and it was also greater than in Haonuoyang. This indicates that Haonuoyang has a stronger antioxidant capacity than Z1 and JG30, while H1 exhibits a higher antioxidant capacity than Nipponbare, JG30, and Haonuoyang. Similar to the changes in superoxide dismutase (SOD) activity, POD activity likely works together with SOD, contributing to the enhanced disease resistance in Haonuoyang and H1, potentially through the scavenging of reactive oxygen species and modulation of plant defense responses.

During the 0–72 h period following PXO99 inoculation, catalase (CAT) activity in Haonuoyang exhibited an increase followed by a decrease. CAT activity sharply increased from 0 to 12 h, peaking at 24 h. In a similar fashion, CAT activity in G30 also rose significantly from 0 to 12 h. On the other hand, CAT activity in Z1 remained relatively stable with little change (Figure 8f). These results indicate that PXO99 infection enhances CAT activity in Haonuoyang and G30. Furthermore, CAT activity in H1, Nipponbare, and G30 increased rapidly from 0 to 12 h and then leveled off between 12 and 48 h. In general, the CAT activity in H1 was higher than in Nipponbare and G30 (Figure 8e). These findings suggest that variations in CAT activity in H1, Haonuoyang, Nipponbare, and G30 may be linked to the disease-resistance gene *OsZFPH*. Catalase is a critical enzyme involved in scavenging reactive oxygen species in plants, which are responsible for breaking down H_2_O_2_ into harmless H_2_O and O_2_. These enzyme systems, therefore, provide a protective mechanism against stress by maintaining reactive oxygen species balance, safeguarding membrane integrity, and enabling plants to endure, alleviate, or resist pathogen stress to some extent.

During the 0–72 h period following PXO99 inoculation, the hydrogen peroxide (H_2_O_2_) content in H1, Haonuoyang, Nipponbare, and G30 showed similar trends. The H_2_O_2_ content initially increased, which was followed by a decrease, peaking at 24 h. In general, the H_2_O_2_ content in Haonuoyang was higher than in G30, and the H_2_O_2_ content in H1 was higher than in the control, Nipponbare. In contrast, H_2_O_2_ content in Z1 remained relatively unchanged (Figure 8g,h). These findings suggest that the defense mechanisms in H1, Haonuoyang, Nipponbare, and G30 were activated. Based on the changes in H_2_O_2_ content over the 0–72 h period after PXO99 inoculation, it can be inferred that the *OsZFPH* gene is involved in rice disease resistance.

### 3.8. Analysis of the Relationship Between OsZFPH and Exogenous Hormones

**Figure 9:**
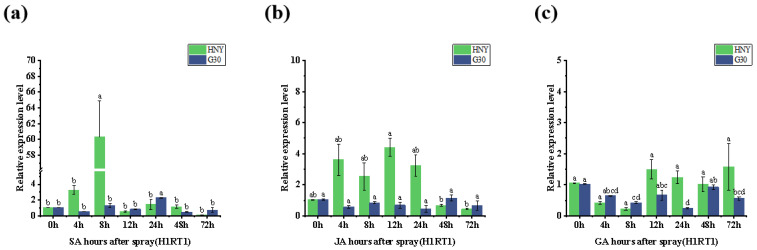
Relative expression of *OsZFPH* at different time points under various hormone treatments in HNY and G30. (**a**) *OsZFPH* expression under salicylic acid (SA) treatment. (**b**) *OsZFPH* expression under jasmonic acid (JA) treatment. (**c**) *OsZFPH* expression under gibberellic acid (GA) treatment. Error bars indicate SD, and different letters denote statistically significant differences (*p* < 0.05).

Following treatment with salicylic acid (SA), jasmonic acid (JA), and gibberellic acid (GA), the expression level of *OsZFPH* was analyzed using real-time PCR. The results revealed that after SA treatment, the gene expression in Haonuoyang was elevated at 4 h and significantly increased at 8 h. In contrast, in the control, Jingang 30, there was a minimal change in gene expression (Figure 9a). After JA treatment, the gene expression in Haonuoyang remained relatively high at 4 h, 8 h, 12 h, and 24 h. Similarly, in the control, Jingang 30, expression levels showed little fluctuation (Figure 9b). Following GA treatment, the relative expression of this gene was increased at 12 h, 24 h, 48 h, and 72 h (Figure 9c). These results suggest that the *OsZFPH* gene is positively regulated by the exogenous hormones SA, JA, and GA.

### 3.9. Subcellular Localization of OsZFPH

**Figure 10:**
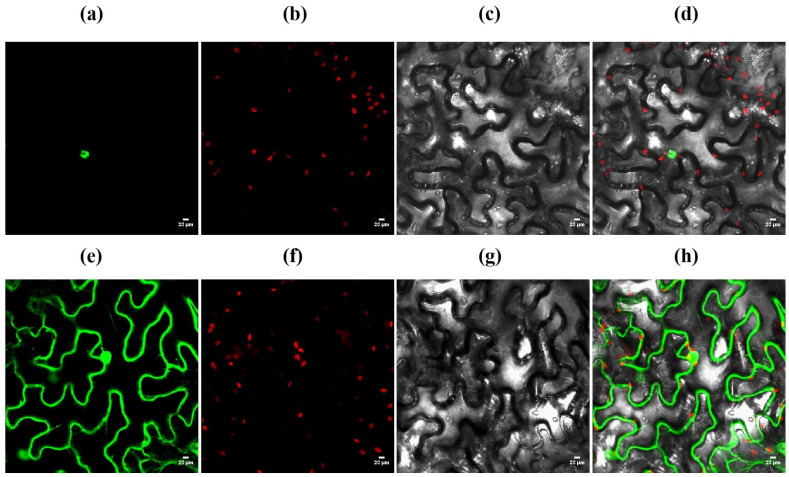
Subcellular localization of *OsZFPH*. (**a**) Target protein fluorescence channel. (**b**) Chloroplast fluorescence channel. (**c**) Bright field. (**d**) Merged image. (**e**) Fluorescent empty vector control. (**f**) Empty vector chloroplast fluorescence channel. (**g**) Empty vector bright field. (**h**) Merged empty vector control. The green dots represent the fluorescence—emitting sites of the target protein in the cell nucleus. The red dots represent the fluorescence emitted by chloroplasts.

To precisely determine the function of *OsZFPH*, a subcellular localization analysis was conducted. Initially, tobacco leaves were infiltrated with *A. tumefaciens* containing the labeled construct (Figure 10). Examination using a laser-scanning confocal microscope subsequently revealed that the fluorescent plasmid, when fused with the *OsZFPH* vector, exhibited strong green fluorescence exclusively in the cell nucleus. The fluorescence signal was distinctly defined and confined specifically to the nuclear region.

### 3.10. Screening for Proteins Interacting with OsZFPH Protein

**Figure 11:**
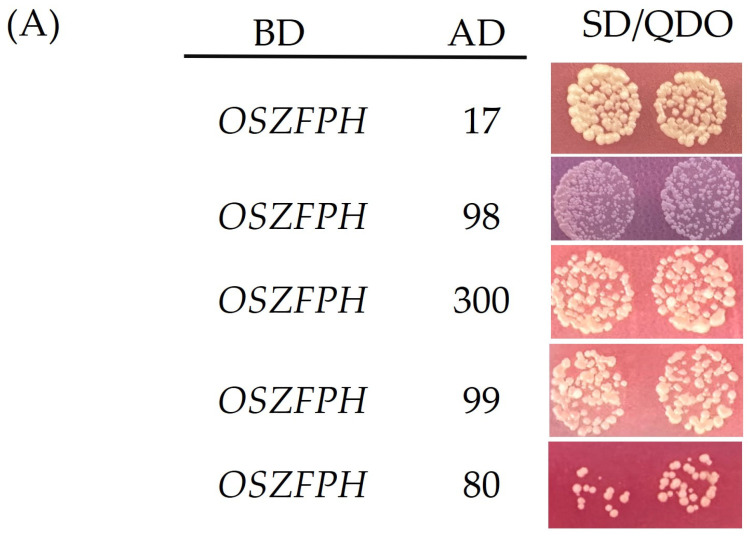

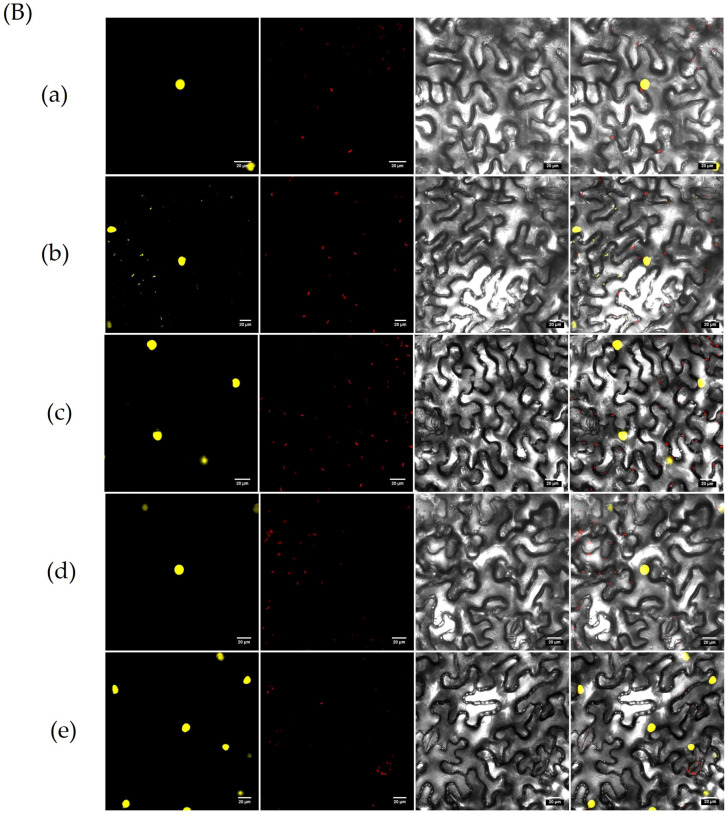
Identification of *OsZFPH*-interacting proteins using yeast two-hybrid (Y2H) and bimolecular fluorescence complementation (BiFC) assays. (**A**) Interaction of *OsZFPH* with five candidate proteins in the Y2H system: XP_015627417.1 (WRKY71, abbreviated as 17), XP_015618098.1 (protein-tyrosine phosphatase PTP1, abbreviated as 98), XP_015637300.1 (peptide-methionine-sulfoxide reductase B3, abbreviated as 300), XP_015635499.1 (phosphatidylinositol-4-kinase, abbreviated as 99), and XP_015633380.1 (gibberellin 2-β-dioxygenase, abbreviated as 80). (**B**) Verification of *OsZFPH*–protein interactions using BiFC: (**a**) XP_015635499.1 (phosphatidylinositol-4-kinase γ6), (**b**) XP_015637300.1 (peptide-methionine-sulfoxide reductase B3), (**c**) XP_015633380.1 (gibberellin 2-β-dioxygenase), (**d**) XP_015618098.1 (protein-tyrosine phosphatase PTP1), and (**e**) XP_015627417.1 (WRKY71). Each row presents images in the fluorescence channel, chloroplast fluorescence channel, bright field, and merged image.

Using *OsZFPH* as the bait and five proteins as the prey, vectors were constructed. Yeast two-hybrid assays, as depicted in Figure 11, demonstrated that all five proteins exhibited normal growth on quadruple-dropout plates, confirming their interactions with *OsZFPH*. The interacting proteins included XP_015627417.1 (WRKY71), XP_015618098.1 (protein-tyrosine phosphatase PTP1), XP_015637300.1 (peptide-methionine-sulfoxide reductase B3), XP_015635499.1 (phosphatidylinositol-4-kinase γ6), and XP_015633380.1 (gibberellin 2-β-dioxygenase) (Figure 11A). Further validation through Bimolecular Fluorescence Complementation (BiFC) confirmed that XP_015635499.1 (phosphatidylinositol-4-kinase γ6), XP_015637300.1 (peptide-methionine-sulfoxide reductase B3), XP_015633380.1 (gibberellin 2-β-dioxygenase), XP_015618098.1 (protein-tyrosine phosphatase PTP1), and XP_015627417.1 (WRKY71) interacted with *OsZFPH* in tobacco leaves with localization observed specifically in the nuclear region (Figure 11B).

### 3.11. Haplotype Analysis Analysis of the Transcription Factor OsZFPH in Different Rice Species Materials

**Figure 12:**
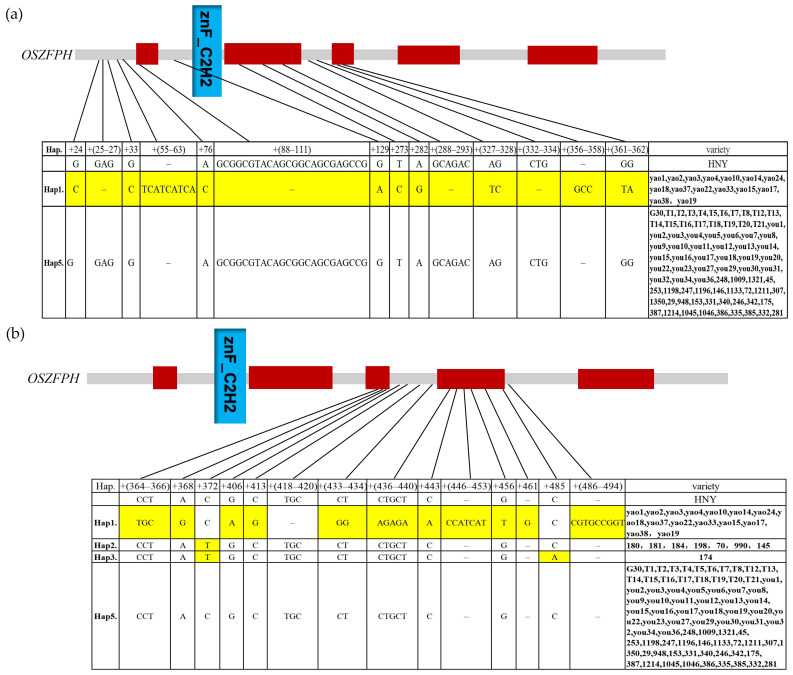

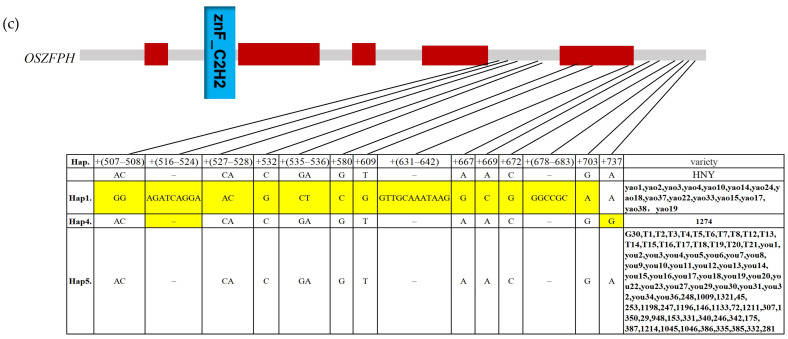
Haplotype analysis of OsZFPH in different rice accessions. (**a**) Haplotype analysis of the structural domains of the *OsZFPH* gene (0–362 bp) in 104 rice accessions. (**b**) Haplotype analysis of the structural domains of the *OsZFPH* gene (364–494 bp) in 104 rice materials. (**c**) Haplotype analysis of the structural domains of the *OsZFPH* gene (507–737 bp) in 104 rice materials. T: *O. rufipogon* population; HNY: Haonuoyang; you: *O. granulata* population; yao: *O. officinalis* population; numerals represent resequenced rice accessions. The blue area represented the C_2_H_2_ domain, and the red area represented the complex domains. The yellow markings indicated the base differences between different haplotypes.

Haplotype analysis was performed on the *OsZFPH* gene domain sequences of 104 rice varieties, including 15 *O. officinalis*, 29 *O. granulata*, 18 *O. rufipogon*, 40 resequenced rice varieties, Haonuoyang, and Jingang 30. Five major haplotypes were identified and tentatively designated as Hap1, Hap2, Hap3, Hap4, and Hap5. In comparison to Haonuoyang, *O. officinalis* exhibited more distinct base insertions, deletions, or mutations, with Hap1 as its predominant haplotype, comprising 15 *O. officinalis* accessions. Hap2 and Hap3 collectively contained eight resequenced accessions, while Hap4 included a single resequenced accession. Hap5 was identical to the Haonuoyang sequence and represented a mixed-resource haplotype, encompassing Jingang 30, 31 resequenced rice varieties, 18 *O. rufipogon*, and 29 *O. granulata* (Figure 12 and Tables S1 and S2).

## 4. Discussion

This study represents the first report on the isolation of the *OsZFPH* gene in rice through homologous sequence amplification, confirming that the *OsZFPH* coding sequence encodes a C_2_H_2_-type zinc finger protein, which plays a crucial role in Haonuoyang’s resistance to bacterial blight (BB). Overexpression of this gene enhanced defense mechanisms in rice by upregulating defense-related genes, increasing antioxidant enzyme activities, and modulating ROS signaling. The interaction of *OsZFPH* with multiple proteins further emphasized its involvement in a complex network of signaling pathways that regulate plant immunity. Hormone signaling analysis highlighted the critical function of *OsZFPH* in the hormone-mediated disease resistance response, while subcellular localization studies linked *OsZFPH* to the direct regulation of gene expression, response to hormone signal transduction, and coordination of disease resistance-related physiological processes.

Zinc finger protein transcription factors are classified into nine subclasses based on the number and arrangement of conserved cysteine and histidine residues in their primary structure as well as differences in their functional roles. These subclasses include C_2_H_2_, C_8_, C_6_, C_3_H_4_, C_2_HC, C_2_H_5_, C_4_, C_4_HC_3_, and C_3_H [23]. C_2_H_2_-type zinc finger proteins are involved in signal transduction pathways. Their zinc finger structure consists of a highly conserved sequence, Cys-X_2–4_-Cys-X_12_-His-X_3–5_-His, where X represents variable amino acids. Each zinc finger forms a stable finger-like structure with two cysteines and two histidines coordinated by Zn^2+^ ions, enabling specific binding to DNA or RNA. The zinc finger domain of OsZFP1 regulates gene expression by binding to GC-rich sequences, such as the GCC-box, in target gene promoters [24]. The identified TFIIIA-type zinc finger proteins in rice possess a double zinc finger structure and are associated with stress responses [25,26,27,28]. More than 30 C_2_H_2_-type zinc finger proteins in rice have been recognized for their role in disease resistance with over half responding to rice blast and bacterial blight [29]. In this study, the newly cloned zinc finger protein gene *OsZFPH* from the local rice variety Haonuoyang exhibited the unique QALGGH motif characteristic of zinc finger proteins. Additionally, it contained a nuclear localization signal region (RRDRAKLR, amino acids 77–84) and an EAR-motif transcriptional regulatory region (VLDLEL, amino acids 227–232) but lacked an L-box region, classifying it as a typical TFIIIA-type zinc finger protein. Phylogenetic analysis demonstrated a high degree of conservation of *OsZFPH*, showing similarity to the transcription factor RIBBIT EARS (LOC9271775) in indica rice, suggesting that OsZFPH may also play a vital role in rice growth.

### 4.1. OsZFPH Enhances Rice Resistance to Bacterial Blight via JA/SA/GA Signaling and Antioxidant Enzyme-Mediated ROS Homeostasis

JA and SA are two plant hormones involved in host–pathogen interactions. JA primarily functions as a defense signaling molecule, responding to plant stress caused by pathogens and other environmental adversities. Through signal transduction, it modulates the expression of specific plant genes, disrupting immune system balance and mediating disease resistance responses [30]. The C_3_H-type zinc-finger protein C_3_H_12_ positively regulates rice resistance to Xoo by promoting JA accumulation and activating JA-signaling genes. Silencing or knocking out C_3_H_12_ reduces JA levels and increases susceptibility to Xoo, highlighting its role in JA-mediated disease resistance [31]. Additionally, research has indicated that allene oxide synthase (AOS) is essential for JA biosynthesis. In the rice genome, four genes encode AOS enzymes, among which OsAOS1 and OsAOS2 are active and enhance resistance to Magnaporthe oryzae [32]. WRKY13 activates the SA signaling pathway and regulates the expression of SA-related genes [33]. SA plays a crucial role in plant defense by activating related pathways and genes. Knockout studies of key genes involved in SA biosynthesis, such as ICS1, have demonstrated that endogenous SA is essential for basal resistance, influencing callose deposition and cell wall reinforcement [21,34]. Moreover, plant viruses suppress DELLA-mediated broad-spectrum resistance by hijacking the gibberellin (GA) receptor, indicating that the GA signaling pathway is a critical regulatory node in disease defense. Several unrelated viral effectors target the GA receptor OsGID1 to destabilize *SLR1*, thereby disrupting crosstalk with JA signaling and weakening JA-mediated antiviral responses [35].

In this study, it was observed that following the application of exogenous hormones, the expression of key genes such as *LOX*, *AOS1* (in the JA pathway), and PAL (in the SA pathway) was higher in *OsZFPH*-overexpressing plants compared to knockouts. These findings suggest that upon Xoo invasion, OsZFPH regulates genes encoding OsLOX, OsAOS, and OsPAL, thereby activating JA, SA, and GA defense-signaling pathways in rice. This activation induces other resistance-related genes, leading to an enhanced response against bacterial blight.

SOD, POD, and CAT constitute the inherent protective enzyme systems in crops. These enzymes function both independently and cooperatively, playing a crucial role in stress-resistant metabolism [36]. They are widely distributed across organisms and cells, contributing significantly to stress resistance. CAT and POD decompose H_2_O_2_ into H_2_O and O_2_, which is essential for the removal of reactive oxygen species (ROS). SOD catalyzes the conversion of superoxide anions into H_2_O_2_ and O_2_, making it a key component in antioxidation. Collectively, these enzymes form the enzyme protection system [36]. H_2_O_2_, commonly present in plants, can be degraded by these enzymes. However, excessive accumulation may damage cellular components and accelerate cell aging. Research has indicated that rice responses to Xoo are associated with ROS levels. Susceptible rice varieties produce fewer free radicals during pathogen attack, whereas resistant varieties generate higher levels to counteract infection [37].

In this study, plants were inoculated with PXO99, and *OsZFPH* was knocked out for comparison with Haonuoyang. The wild-type strain exhibited higher SOD, POD, and CAT activities, along with increased H_2_O_2_ levels, compared to the knockout strain. A similar trend was observed when OsZFPH was overexpressed in Nipponbare, suggesting that OsZFPH influences physiological and biochemical processes, thereby enhancing disease resistance in rice. Additionally, these findings confirm that *OsZFPH* contributes to improving the antioxidant capacity of rice, strengthening disease resistance, and maintaining normal physiological metabolism in rice leaves.

### 4.2. OsZFPH Interacted with WRKY71, PTP1, and Metabolic Enzymes to Form a Multi-Layered Defense Network Against Bacterial Blight in Rice

The identification of five proteins interacting with OsZFPH through yeast two-hybrid and BiFC assays provides crucial insights into the molecular mechanisms underlying *OsZFPH*-mediated resistance. WRKY71 in rice inhibits JA signaling by binding to the promoters of disease-resistant genes such as *PR1a* [38,39]. It is possible that WRKY71 forms a transcriptional complex with *OsZFPH*, which then binds to the promoter regions of defense-related genes, co-regulating their expression and enhancing defense responses. Protein-tyrosine phosphatase PTP1 may influence the phosphorylation status of key signaling components in the defense pathway [40]. Since phosphorylation and dephosphorylation are essential regulatory mechanisms in signal transduction, PTP1’s interaction with *OsZFPH* could contribute to fine tuning the defense signaling cascade. Peptide-methionine-sulfoxide reductase B5 likely protects proteins from oxidative damage by reducing methionine sulfoxides back to methionine [41,42], which is a function that is particularly critical during pathogen infection when ROS accumulation can lead to protein oxidation.

Phosphatidylinositol-4-kinase and gibberellin 2-β-dioxygenase are likely involved in lipid-mediated signaling [43] and hormonal regulation [44], respectively. Lipid-mediated signaling plays a crucial role in membrane-associated processes and the activation of defense responses. Excessive PI4P and PI(4,5)P2 disrupt actin cytoskeleton organization and cell elongation with PI(4,5)P2 having a stronger effect on Arp2/3 complex-nucleated actin-branching networks. This suggests that the Arp2/3 complex may be a target of PI(4,5)P2 modulation, linking phosphoinositide homeostasis to plant growth [43]. PI4K might be involved in *OsZFPH*-related membrane signal transduction. Additionally, hormonal regulation plays a vital role in balancing plant growth and defense. GA2ox suppresses growth by degrading active gibberellins, thereby prioritizing resource allocation toward disease resistance [44]. It is possible that GA2ox cooperates with *OsZFPH* to regulate the growth–defense trade-off. The interactions of these proteins with *OsZFPH* suggest a complex network of molecular mechanisms contributing to rice defense against Xoo. Further experimental validation is necessary to confirm these hypotheses.

## 5. Conclusions

In this study, the OsZFPH gene in rice was isolated for the first time using the homologous sequence amplification method. The C_2_H_2_-type zinc finger protein encoded by this gene played a crucial role in enabling Haonuoyang to resist bacterial blight. The overexpression of *OsZFPH* resulted in the upregulation of defense-related genes, increased activities of antioxidant enzymes (SOD, POD, CAT), regulation of reactive oxygen species (ROS) signals, and reinforcement of the rice defense system. Additionally, *OsZFPH* interacted with multiple proteins, positioning itself as a key component in the complex signal transduction pathways associated with plant immunity.

In terms of hormonal regulation, JA, SA, and GA were significantly involved in the disease resistance process in rice. JA functioned as a defense signaling molecule, assisting in pathogen resistance, while JA accumulation regulated disease resistance. SA activated defense-related pathways and genes, whereas the GA signaling pathway played a critical role in disease resistance. The findings demonstrated that following the application of exogenous hormones, expression levels of key genes in the JA and SA pathways were elevated in OsZFPH-overexpressing plants, which likely contributed to the synergistic activation of defense signaling pathways. Furthermore, yeast two-hybrid and bimolecular fluorescence complementation experiments identified five proteins interacting with *OsZFPH*, including WRKY71. These proteins were involved in various biological processes such as transcriptional regulation, signal transduction, and antioxidation. Together with *OsZFPH*, they formed a multilayered defense network against bacterial blight, providing a theoretical foundation for future research on rice disease resistance and breeding applications.

## Figures and Tables

**Table 1:** Gene amplification primers.

Primer Name	Sequences	Purpose
H1-RT-1F	AGCCACCAAGAAGAAGAAGC	qPCR
H1-RT-1R	TGGAGGTGGTGCTAGGTTTAG	qPCR
PR10-1F	CCCTGCCGAATACGCCTAA	qPCR
PR10-1R	CTCAAACGCCACGAGAATTTG	qPCR
PR1b-F	GGCAACTTCGTCGGACAGA	qPCR
PR1b-R	GGCAACTTCGTCGGACAGA	qPCR
OsActin2-F	GAGTATGATGAGTCGGGTCCAG	qPCR
OsActin2-R	ACACCAACAATCCCAAACAGAG	qPCR
LOX-F	AAACGCTCGCTGGCATCAAC	qPCR
LOX-R	ATCGCCTCCTCCACCGTCAT	qPCR
AOS1-F	GGTGAAGAAGGACTACGACCGC	qPCR
AOS1-R	CCGAACGAGTTGAAGCAGAGC	qPCR
PAL-F	CACAAGCTGAAGCACCACCC	qPCR
PAL-R	GAGTTCACGTCCTGGTTGTG	qPCR
H1-1F	ATGGAGGAGCAAAGAGGCGA	PCR
H1-1R	TAAGCGTGCACAACAGCTT	PCR
39425	CAGTGGTCTCACAACATGGAGGAGCAAAGAGGCGAGG	Subcellular localization vector construction
39425	CAGTGGTCTCATACAAGCGTGCACAACAGCTTTAGGAGGAG	Subcellular localization vector construction

Note: The primers of PR10, PR1b, LOX, AOS1, and PAL were all sourced from the known literature, the primers for OsActin2 were designed according to reference [11], while H1 and 39425 were designed by this study independently.

## Data Availability

Data will be made available on request.

## Supplementary Materials

The following supporting information can be downloaded at https://www.mdpi.com/article/10.3390/genes16030240/s1, Table S1: Analysis of *OsZFPH* cDNA haplotype types; Table S2: Rice varieties used in this experiment.
